# Heat Shock Protein–Peptide and HSP-Based Immunotherapies for the Treatment of Cancer

**DOI:** 10.3389/fimmu.2016.00171

**Published:** 2016-04-29

**Authors:** Maxim Shevtsov, Gabriele Multhoff

**Affiliations:** ^1^Department of Radiation Oncology, Klinikum rechts der Isar, TU München, Munich, Germany; ^2^Institute of Cytology of Russian Academy of Sciences (RAS), St. Petersburg, Russia

**Keywords:** HSP70 heat shock proteins, HSP90 heat shock proteins, cancer vaccine, innate immunity, adaptive immunity

## Abstract

Intracellular residing heat shock proteins (HSPs) with a molecular weight of approximately 70 and 90 kDa function as molecular chaperones that assist folding/unfolding and transport of proteins across membranes and prevent protein aggregation after environmental stress. In contrast to normal cells, tumor cells have higher cytosolic heat shock protein 70 and Hsp90 levels, which contribute to tumor cell propagation, metastasis, and protection against apoptosis. In addition to their intracellular chaperoning functions, extracellular localized and membrane-bound HSPs have been found to play key roles in eliciting antitumor immune responses by acting as carriers for tumor-derived immunogenic peptides, as adjuvants for antigen presentation, or as targets for the innate immune system. The interaction of HSP–peptide complexes or peptide-free HSPs with receptors on antigen-presenting cells promotes the maturation of dendritic cells, results in an upregulation of major histocompatibility complex class I and class II molecules, induces secretion of pro- and anti-inflammatory cytokines, chemokines, and immune modulatory nitric oxides, and thus integrates adaptive and innate immune phenomena. Herein, we aim to recapitulate the history and current status of HSP-based immunotherapies and vaccination strategies in the treatment of cancer.

## Background

Heat shock proteins (HSPs) with the molecular weights of approximately 70 and 90 kDa have the capacity to stimulate antitumor immune responses either as carriers for antigenic peptides, which can be cross-presented by major histocompatibility complex (MHC) class I molecules, or as natural immunogens (1–3). Depending on the availability of ATP and ADP, members of the heat shock protein 70 (HSP70) family have the capacity to release and bind tumor-specific antigens, respectively. Following cross-presentation on MHC class I antigens, a CD8+ cytotoxic T cell response is initiated. Preclinical models revealed that vaccination with HSP–peptide complexes purified from tumor, but not normal cells, are able to mediate specific and protective immunity against autologous tumors. In recent years, a large number of receptors, including the alpha-2 macroglobulin receptor CD91 (4), lectin-like oxidized low-density lipoprotein receptor-1 (LOX-1) (5, 6), scavenger receptor expressed by endothelial cells-1 (SREC-1) (7), toll-like receptors-2/4 (TLRs-2/4) (8–10), their cofactors CD14 (11, 12), fasciclin EGF-like, laminin-type EGF-like and link domain-containing scavenger receptor-1 (FEEL-1), common lymphatic endothelial and vascular endothelial receptor-1 (Clever-1), stabilin-1 (Stab-1), and CD40 (13), have been found to be involved in the uptake of HSPs and HSP-chaperoned exogenous peptides into antigen-presenting cells (APCs).

Despite their high degree of homology certain HSP sequences are not conserved and thus act as immunogenes, which can be recognized as foreign by the host’s innate and adaptive immune system, especially when they are presented in a tissue/tumor-specific manner (14). In combination with pro-inflammatory cytokines, including interleukin 2 (IL-2), IL-12, or IL-15, the major stress-inducible Hsp70 (HSPA1A) or non-conserved sequences derived thereof has been found to activate the cytolytic, proliferative, and migratory capacity of natural killer (NK) cells (15). This activation was accompanied by an upregulated expression density of activatory C-type lectin receptors CD94/NKG2C and NKG2D on NK cells (16). Furthermore, an intra-tumoral infusion of free, recombinant Hsp70 has been shown to increase the infiltration of NK cells and CD8+ cytotoxic T cells into tumors and the secretion of interferon gamma (IFN-γ) (17, 18).

## HSP–Peptide Complexes as a Vaccine Strategy

In 1986, the group of Old et al. firstly described a glycoprotein with a molecular weight of 96 kDa (gp96), which was found to act as a tumor rejection antigen. Gp96 was isolated from mouse fibrosarcomas that were chemically induced by the carcinogen methylcholanthrene A (1). Li and Srivastava (19) characterized gp96 as an ER-residing member of the HSP90 family, which contains an ATPase activity. In addition to gp96, members of the HSP70 family that also possess an ATPase domain appeared to be equally immunogenic for the adaptive immune system such as gp96 when tumor-specific antigens were bound to them (20, 21). Since ATP sepharose columns are widely used for the purification of HSP–peptide complexes from tumor cells, there is a risk that the immunogenic peptides are dissolved from the HSP–peptide complexes during purification due to their ATPase activity (22). Therefore, a novel purification method, which was based on ADP-affinity chromatography, has been established for the isolation of intact HSP–peptide complexes (23).

Immunogenic peptides chaperoned by gp96 and HSP70s were not only found to elicit specific immune responses against cancer (24, 25) but also against infectious diseases (26, 27), indicating the broad applicability of HSP-based vaccines. It was also found that following oxidative stress the immunogenicity of HSP-based vaccines was found to be increased (28). This finding might be explained, on the one hand, by the fact that the amount and the repertoire of immunogenic peptides might differ in stressed and non-stressed cells. On the other hand, it is possible that stress-inducible members of HSP families might be better qualified for chaperoning immunogenic peptides than their constitutively expressed correlates. Since HSP-chaperoned peptides only mediate protective immunity against autologous, but not allogeneic tumors (24), and HSP–peptide complexes eluted from healthy tissues were found to be inefficient in stimulating T cell-mediated immunity and was assumed that HSP-chaperoned peptides are tumor cell type specific.

Furthermore, an efficient rejection of tumors in preclinical models requires the presence of CD8+ T cells in the priming phase and that of CD4+ helper, CD8+ cytotoxic T cells, and M1 macrophages in the effector phase (21). Exogenous antigens, which are typically presented by MHC class II antigens, can be channeled by HSPs into the endogenous pathway and thus can be presented on MHC class I molecules (29). This HSP-mediated switch of peptides from the endogenous MHC class II to the MHC class I pathway is also termed as antigen cross-presentation (29–31).

For a while, the mechanism how exogenous HSP–peptide immune complexes are taken up by APCs remained elusive because HSP-specific receptors had not been identified and characterized. The group of Binder et al. classified the interaction of HSP–peptide complexes with APCs as specific and saturable. These attributes are typical for a receptor–ligand interaction (32). The same group was among the first who identified CD91 as a receptor for immunogenic peptides complexed with HSP90 and HSP70 families and for calreticulin (33). CD91, which is also termed low-density lipoprotein-related protein, was initially described as a receptor for alpha-2 macroglobulin (4). Until today, a large variety of different receptors, such as LOX-1 (6), SREC-1, FEEL-1, Clever-1, Stab-1 (5, 7, 34, 35), TLRs-2/4, and their cofactor CD14 (11, 12, 36) and CD40 (13), have been shown to be involved in the uptake and signaling of HSP70 and HSP90 complexes with APCs (37).

It is important to note that the capacity of HSPs or HSP–peptide complexes to elicit antitumor-specific immunity is highly dependent on the dose. Although low doses of HSP–peptide complexes have been found to be efficient in the stimulation of antitumor immune responses, a 5- to 10-fold higher dose than the optimal stimulatory dose turned out to be ineffective or even immunosuppressive (38). High doses of gp96–peptide complexes were found to induce immune tolerance and thus were applied to treat autoimmune diabetes and encephalomyelitis in preclinical models (39, 40). The mechanisms, which are involved in the induction of tolerance by HSP70s, have been found to be associated with TLR2 and TLR4. The TLR2/MyD88 signaling pathway, which is induced after binding of exosomal Hsp70 to TLRs, has been found to mediate protection of the myocardium against ischemic reperfusion injury (10), and the TLR4/ERK1,2/p38/MAPK pathway has been found to initiate pStat3-mediated immunosuppressive activity in myeloid-derived suppressor cells (9).

Based on the knowledge on the molecular characteristics and functions of HSPs and HSP–peptide-based vaccines, the stimulation of antitumor immune responses initiated clinical applications (41). Between 2000 and 2014, gp96 and HSP70–peptide-based vaccines derived from autologous tumor lysates were clinically applied in phase I to phase III clinical trials in different tumor entities including late stage melanoma (42) either alone or in combination with GM-CSF and IFN-γ (43–48), metastatic colon carcinoma (49), renal cell carcinoma (50), gastric carcinoma (51, 52), pancreatic carcinoma (53), chronic myeloid leukemia (54), and glioblastoma (55) (Table 1). The outcome of these trials showed the induction of immunological responses in a large number of patients treated with HSP–peptide complexes; however, clinical responses (CRs) were observed only in certain patient subgroups.

**Table 1:** **Phase I–III clinical trials using HSP-based vaccines**.

HSP vaccine	Tumor entity	Study	Reference
Gp96	Late stage melanoma	Pilot	(42)
Gp96 + sPD-1	Malignant melanoma	Phase I–III	(46–48)
Gp96 + GM-CSF	Malignant melanoma	Phase I–II	(44)
Gp96 + GM-CSF + IFN	Malignant melanoma	Phase I–II	(45)
Hsp70	Malignant melanoma	Phase I	(44)
Gp96	Metastatic colon carcinoma	Phase I	(49)
Gp96	Gastric carcinoma	Phase I	(51, 52)
Gp96	Pancreatic carcinoma	Phase I	(53)
Gp96	Hodgkin lymphoma	Phase I	(54)
Hsp70	Chronic lymphatic leukemia	Phase I	(84)
Hsp70	Advanced solid tumors	Pilot	(76)
Gp96	Glioblastoma	Phase I–II	(55)
Hsp70	Glioblastoma	Phase I	(76)
Hsp70-activated NK cells	Colon carcinoma, NSCLC	Phase I–II	(72, 73)
Hsp70	HIV	Phase I	(26)
Hsp70 mRNA	HCC	Phase I	(85)

## HSP70 in the Stimulation of Innate Immunity

Heat shock protein 70 has been found to be overexpressed in tumor cells. Hsp70 is presented on the cell membrane of a large variety of solid tumors, including lung, colorectal, breast, squamous cell carcinomas of the head and neck, prostate and pancreatic carcinomas, glioblastomas, sarcomas, and hematological malignancies, but not on corresponding normal tissues (56, 57). A membrane Hsp70+ phenotype has been determined either directly on single cell suspensions of freshly isolated tumor biopsies by cell surface iodination/biotinylation (58, 59) and flow cytometry using cmHsp70.1 monoclonal antibody (60) or indirectly in the serum of patients using a novel lipHsp70 ELISA (61). In contrast to commercially available ELISA systems, the lipHsp70 ELISA specifically detects free and lipid-bound, exosomal Hsp70 which is actively released by viable tumor cells. Therefore, it is assumed that the quantification of exosomal Hsp70 in the serum serves as a measure for viable tumor mass in a patient and thus might provide a diagnostic/prognostic biomarker in the future (62). A membrane Hsp70+ tumor phenotype has been found to be associated with highly aggressive tumors, causing invasion and metastases and resistance to cell death (57, 63, 64). However, NK cells, but not T cells, were found to kill membrane Hsp70+ tumor cells after preactivation with naturally occurring Hsp70 or an Hsp70–peptide (TKD) derived thereof in combination with low dose IL-2 (TKD/IL-2) (65). Since the induction of the cytolytic activity of NK cells with Hsp70–peptide is dose dependent and saturable, it was assumed that the interaction of NK cells with the peptide might also be receptor mediated. By antibody and protein/peptide blocking assays, the C-type lectin receptor CD94 was identified as a potential receptor that mediates the interaction of NK cells with Hsp70–peptide. CD94 forms a heterodimer either with the coreceptor NKG2A or NKG2C and thus can act as an inhibitory or activation receptor complex (66–69). Following incubation of NK cells with Hsp70 protein or Hsp70–peptide + IL-2, the density of CD94 was found to be upregulated concomitant with an increased cytolytic and migratory activity against membrane Hsp70+ tumor cells (70). In addition, also other activatory NK cell receptors, such as NKG2D, and natural cytotoxicity receptors (NCRs), but not inhibitory killer-cell immunoglobulin-like receptors (KIRs), were found to be upregulated on NK cells upon stimulation with Hsp70–peptide + IL-2.

**Figure 1:**
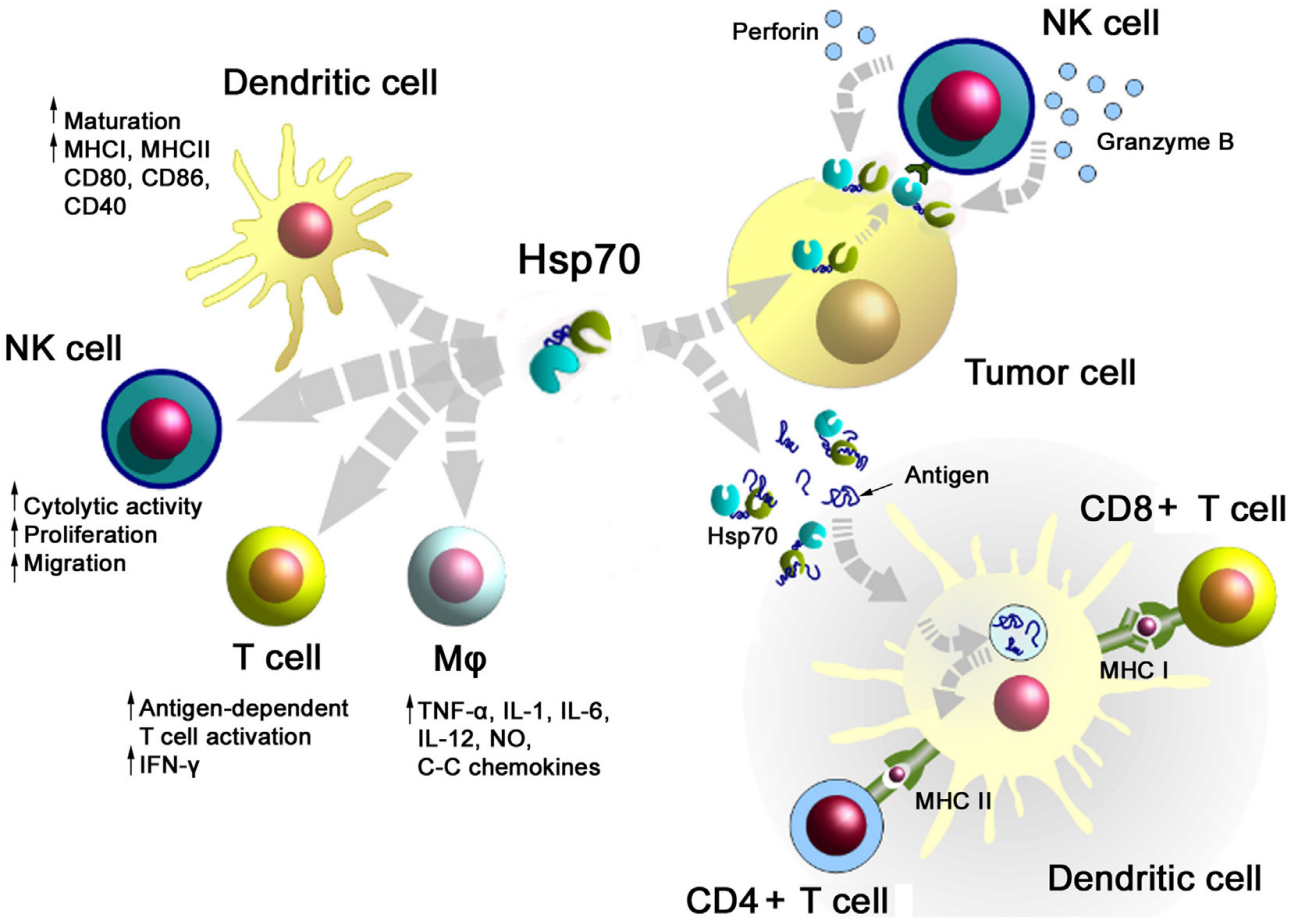
**Major immune modulatory functions of heat shock protein 70 (Hsp70) either alone bound to exosomes or in combination with tumor-derived peptides**. Abbreviations: IFN-γ, interferon gamma; IL, interleukin; Mφ, macrophages; MHC, major histocompatibility complex; NK cells, natural killer cell; NO, nitric oxide.

A summary of major activities of Hsp70 in inducing adaptive and innate antitumor immune responses is illustrated in Figure 1. On the one hand, Hsp70 either alone or in combination with immunogenic peptides is able to induce the maturation of dendritic cells (DCs), activate the cytolytic, proliferative, and migratory capacity of NK cells, stimulate the antigen-dependent T cell activation and IFN-γ secretion, induce the release of pro- and anti-inflammatory cytokines, on the other hand, membrane-bound Hsp70 acts as a tumor-specific antigen, which is recognized by preactivated NK cells. As a carrier for HSP-chaperoned tumor-specific antigens members of the HSP70 and HSP90 family have been found to support antigen uptake, processing, and presentation on MHC class I to CD8+ cytotoxic T lymphocytes and on MHC class II molecules to CD4+ helper T cells.

The mechanism how Hsp70 preactivated NK cells lyse membrane Hsp70+ tumor cells could be identified as granzyme B-mediated apoptosis. The cell death-inducing serine protease granzyme B has been found to directly interact with membrane Hsp70 on tumor cells, as determined by different methods including matrix-laser desorption ionization time to flight mass peptide finger printing (MALDI-TOF), Western blot, and flow cytometry (71). NK cells that have been prestimulated with Hsp70–peptide + IL-2 showed a significantly upregulated production of granzyme B, whereas the intracellular levels of perforin were found to be upregulated only moderately (16, 70). Since tumor cells that lack an Hsp70 membrane expression are not lysed by granzyme B, as demonstrated in isogenic tumor cell systems that differ in their membrane Hsp70 expression levels, it was concluded that Hsp70–peptide + IL-2 preactivated NK cells predominantly kill their target cells *via* granzyme B-mediated apoptosis (71).

Safety and tolerability of *ex vivo* TKD/IL-2 stimulated, autologous NK cells have been demonstrated in patients with metastasized colorectal and NSCLC in a Phase I clinical trial (72). Based on promising clinical results of this Phase I trial, a Phase II randomized clinical study was initiated in 2015 (73). The primary objective of this multicenter proof-of-concept trial is to examine whether an adjuvant treatment of NSCLC patients after platinum-based radiochemotherapy (RCT) with Hsp70–peptide TKD + IL-2-activated, autologous NK cells is clinically effective. Only membrane Hsp70+ tumor patients will be recruited into the trial since membrane Hsp70 was identified as the tumor-specific target for Hsp70–peptide + IL-2 preactivated NK cells. The primary endpoint of this study is the progression-free survival that will be compared between patients who received RCT or RCT+ an NK cell-based immunotherapy. As secondary endpoints overall survival, toxicity, quality-of-life, and biological responses will be determined in both study groups.

## HSPs as Adjuvants for the Stimulation of Antitumor Immune Responses

Heat shock proteins, especially the major stress-inducible Hsp70, can provide cytokine function, which initiate both, innate and adaptive immunity (74–77). In parallel, these HSPs can act as classical chaperones that facilitate uptake, processing, and presentation of tumor antigens into APCs. Moreover, exogenously delivered, purified Hsp70 was shown to sensitize cancer cells to lymphocyte-mediated cytotoxicity due to triggering the translocation of its intracellular analog to the tumor cell surface and due to an increased release of Hsp70 into the extracellular milieu (18). For these reasons, the aforementioned immunomodulatory activities of Hsp70 have been widely exploited for therapeutic approaches in recent years either as single treatment or in combination with other treatment modalities to generate an effective antitumor immunity. The intra-tumoral injection of Hsp70 protein or an upregulation of Hsp70 within the tumor by an *hsp70.1* gene transfer was shown to have a significant therapeutic potential in preclinical studies (18, 78–81). Thus, prolonged intra-tumoral delivery of exogenous Hsp70 in a rat glioblastoma model caused a significant inhibition of tumor progression, which as accompanied by an increased cytotoxic activity of NK cells and CD8+ T lymphocytes (82). A comparable therapeutic efficacy was previously reported by Rafiee et al. (79) who showed a complete tumor eradication following transfection of the *hsp70.1* gene sequence into mouse tumor cells. The systemic antitumor immune response was found to be mediated by CD4+ and CD8+ T cells (79). Presumably, a combination of Hsp70-based therapies with other immunological approaches, such as immune- and T cell check-point inhibitors, might further increase the therapeutic efficacy. In another approach, the intra-tumoral injection of Hsp70 was combined with mild local hyperthermia and magnetite cationic liposomes (MCLs). This strategy demonstrated great potential in the treatment of mouse melanoma (81). With regard to these results, our group coupled Hsp70 to nanocarriers such as superparamagnetic iron oxide nanoparticles (SPIONs) (83). Hsp70-SPIONs were shown to effectively deliver immunogenic peptides from tumor lysates to DCs and thus stimulated a tumor-specific, CD8+ cytotoxic T cell response in experimental glioma models (83). Up-to-date several clinical trials clearly demonstrate that the application of Hsp70 either as a single treatment regimen or in combination with other therapies is feasible and can stimulate antitumor immunity in patients (18, 84, 85). Clinical efficacy could be demonstrated in patients with brain tumors who received surgery and intra-tumoral delivery of recombinant Hsp70. Out of 12 patients with late stage brain tumors, one patient showed a complete CR and another patient showed a partial response (PR) (18). The CRs were accompanied by an enhanced Th_1_-cell-mediated immune response and a reduction of immunosuppressive Treg cells. In the Phase I clinical trial reported by Maeda et al., DCs transfected with Hsp70 mRNA (HSP70-DCs) were applied in 12 patients with non-resectable or recurrent HCV-related hepatocellular carcinoma (HCC) (85). The authors demonstrated that 7 out of 12 patients had either a CR or stable disease (SD), suggesting the efficacy of the proposed therapy. In another study, an intra-tumoral vaccination with recombinant oncolytic type-2 adenovirus that overexpresses Hsp70 was found to inhibit primary and metastatic tumors *via* an enhanced oncolytic activity and Hsp70-mediated immune responses (84). Presumably, multimodality tumor-directed therapy based on HSPs in combination with radio, chemo, and/or hyperthermia (86) therapy can be a treatment option for further clinical trials.

## Summary

Heat shock proteins and especially members of the HSP70 and HSP90 families have been found to elicit protective antitumor immunity in preclinical models and in tumor patients either alone or in complex with tumor-derived peptides. HSPs and HSP–peptide complexes can act as typical tumor-specific foreign antigens, chaperokines, and adjuvants that facilitate uptake, processing, and presentation for tumor-specific antigens which are cross-presented by APCs to CD8+ cytotoxic T lymphocytes. Uptake of HSPs and HSP–peptide complexes is mediated by a large variety of different receptors. Depending on the dose of the HSP-based vaccine either immunosuppressive or immunostimulatory activities can be elicited.

## Author Contributions

MS wrote the paragraph about HSPs as an adjuvant and produced the figure; GM wrote the MS.

## Conflict of Interest Statement

The authors report no conflicts of interest. The authors alone are responsible for the content and preparation of this paper.
